# Open Cholecystectomy among Patients undergoing Laparoscopic Cholecystectomy in a Tertiary Care Centre: A Descriptive Cross-Sectional Study

**DOI:** 10.31729/jnma.7371

**Published:** 2022-05-31

**Authors:** Gaurav Katwal, Yeshika Thapa, Aisha Shrestha, Abhishek Bhattarai, Kishor Kumar Tamrakar, Harish Chandra Neupane

**Affiliations:** 1 Department of Surgery, Chitwan Medical College and Teaching Hospital, Bharatpur-10, Chitwan, Nepal

**Keywords:** *cholecystectomy*, *cholelithiasis*, *laparoscopic cholecystectomy*

## Abstract

**Introduction:**

In the current era, laparoscopic cholecystectomy is the treatment of choice for symptomatic gallstone disease. The aim of this study is to find out the prevalence of open cholecystectomy among patients undergoing laparoscopic cholecystectomy in a tertiary care centre.

**Methods:**

It is a descriptive cross-sectional study done among 345 patients at the Department of Surgery of a tertiary care centre from June, 2020 to May, 2021 after receiving ethical clearance from the Institutional Review Committee (Reference number: 0770798-271). Convenience sampling was done. Successive patients who underwent elective laparoscopic cholecystectomy during the study period were included. Standard 4 port laparoscopic technique was used for the laparoscopic cholecystectomy and sub-costal Kocher incision was used for the open cholecystectomy respectively. After data collection, entry and analysis were done in Microsoft Excel 2016. Point estimate at 95% Confidence Interval was calculated along with frequency and proportion for binary data.

**Results:**

Out of 345 patients, the prevalence of open cholecystectomy among patients undergoing laparoscopic cholecystectomy was 6 (1.73%) (0.35-3.11 at 95% Confidence Interval).

**Conclusions:**

This study showed that the prevalence of open cholecystectomy among patients undergoing laparoscopic cholecystectomy was lower when compared to similar studies conducted in similar settings.

## INTRODUCTION

Laparoscopic Cholecystectomy (LC) is the gold standard surgery for symptomatic cholelithiasis with low mortality and morbidity.^1,2^ However, 1% to 15% of conversion rate to Open Cholecystectomy (OC) during laparoscopic cholecystectomy has been reported.^3^ Converted cases were associated with increased perioperative time, complication rates, perioperative costs, the length of hospital stay, and a higher 30-day readmission rate.^3,4^

Conversion was also associated with complications including bile leak, bile duct injury, or bleeding, requiring reoperation or transfusion, and death. A recent detailed critical review found that preoperative variables like male gender, older age, high body mass index, previous abdominal surgery, the severity of cholecystitis, and gallbladder wall thickness were associated with the higher rate of conversion to OC.^4^ However, data regarding its prevalence lacking in our setting.

The aim of this study is to find out the prevalence of open cholecystectomy among patients undergoing laparoscopic cholecystectomy in a tertiary care centre.

## METHODS

It was a descriptive cross-sectional study conducted among 345 patients at the Department of Surgery of Chitwan Medical College and Teaching Hospital from June, 2020 to May, 2021 after receiving ethical clearance from the Institutional Review Committee (Reference number: CMC-IRC/0770798-271). Convenience sampling was done. All the cases of elective LC admitted during the study period were included. Age <10 years, gall bladder malignancy, adults with preoperative choledocholithiasis, and perforated gall bladder were excluded. The sample size was calculated using the formula,

n = (Z^2^ × p × q) / e^2^

  = (1.96^2^ × 0.049 × 0.951) / 0.03^2^

  = 199

Where,

n = minimum required sample size
Z = 1.96 at 95% Confidence Interval (CI)
p = prevalence of open cholecystectomy among patients undergoing laparoscopic cholecystectomy, 4.9%^5^
q = 1-p
e = margin of error, 3%

A minimum required sample size of 199 was calculated. However, a sample size of 345 was taken and convenience sampling was done. Criteria for the diagnosis of symptomatic cholelithiasis are in accordance with the European Association for the Study of the Liver (EASL) Guideline 2016.^6^ LC was performed by the surgeons with an experience of more than 5 years in LC. Standard 4 port laparoscopic technique was used for the laparoscopic cholecystectomy and sub-costal Kocher incision was used for the open cholecystectomy respectively. Informed consent was obtained from every patient before enrollment into the study.

Data regarding demographic details, previous attack of biliary pathology, underlying condition, Huang classification of the biliary system,^7^ and outcome were collected. The data were entered and analysed in Microsoft Excel 2016. Point estimate at a 95% Confidence Interval was calculated along with frequency and percentages for binary data.

## RESULTS

Out of 345 patients, the prevalence of open cholecystectomy among patients undergoing laparoscopic cholecystectomy was 6 (1.73%) (0.353.11 at 95% Confidence Interval). The study showed a female preponderance with a M:F ratio of 1:4 was seen.

The majority of the patients 5 (83.33%) were between the age of 20 and 60 years (Table 1).

**Table 1:** Demographic profile of the patients and conversion rate (n = 6).

Variables		n (%)
**Sex**	Males	2 (33.33)
	Females	4 (66.67)
**Age**	20-60	5 (83.33)
	>60	1 (16.67)
**Previous attack**	No	3 (50.00)
	Yes	3 (50.00)

In total of 6 patients undergoing OC, males were 2 (33.33%) and females were 4 (66.67%) in number. Three (50.00%) of them had dense adhesion at the Calot's triangle with a previous attack of cholecystitis and biliary pancreatitis. One (16.67%) of them had acute calculus cholecystitis with concomitant Common Bile Duct (CBD) stone and adhesion at the Calot's triangle. Another one (16.67%) had Mirrizi syndrome type I and finally, the last one (16.67%) had the aberrant insertion of the right hepatic duct into the cystic duct. One (16.67%) patient had a Huang type A5 biliary system (Table 2).

**Table 2:** Underlying conditions in the patients undergoing open cholecystectomy (n = 6).

Underlying Condition	n (%)
Frozen Calot's triangle	3 (50.00)
CBD stones	1 (16.67)
Mirrizi's syndrome	1 (16.67)
Anomalous anatomy	1 (16.67)

## DISCUSSION

Globally as well as in Nepal, Gall Stone Disease (GSD) is one of the most common biliary diseases.^8^ Symptomatic GSD is problematic and poses a huge economic burden to patients as well as to the country.^9^ Cholecystectomy is one of the most common abdominal surgical procedures performed today. Over 80% is performed laparoscopically in Europe and United States of America (USA).^10^ Different studies have shown that the conversion to an open procedure was affected by multiple aspects like the patient factor, pathology of the gallbladder, and the surgeon factors.^11^

Compared to the previous study from our centre, the average number of total patients decreased from 290 to 245. The overall prevalence of open cholecystectomy among patients undergoing laparoscopic cholecystectomy in the current study was better (1.74%) than in another similar study (4.61%) conducted in the same setting.^12^ This could be due to the ongoing worldwide COVID-19 crisis that led to the overall decrease in elective surgeries in our centre. In the subgroup analysis of the present study, we found equal rates of conversion with a history of the previous attacks of cholecystitis/biliary pancreatitis among the six patients. Also, the conversion to open cholecystectomy was more common among females. A study reported a rate of conversion (1.86%) in a similar study done at another centre in Nepal.^13^ They also found frozen Calot's triangle as the leading underlying conversion to open procedure.

Our results are consistent with a study where the prevalence of open cholecystectomy among patients undergoing laparoscopic cholecystectomy was less than 5% and the male gender was the only risk factor.^1^ Similarly, a systematic review endorsed a convincing association between risk factors like as male sex, older age, high Body Mass Index (BMI), the presence of acute cholecystitis to the conversion of LC.^3^ Thicken gall-bladder wall (4-5 mm), a contracted gallbladder, age >60, male gender, and acute cholecystitis have been reported as risk factors for the conversion.^4^ Contrary to this only the male gender and the history of previous attacks could have led to higher CR in our study. This may be due to less number of patients with thickened GB walls and contracted GB who underwent LC. However, studies have shown that more surgical experience and high surgery volume might not be associated with a lower prevalence of conversion to OC.^14^

In order to prevent conversion to open procedures, different measures have been implemented. A recent systematic review advises focusing on proper dissection techniques along with the basic principles of biliary surgery, to achieve a Critical View of Safety (CVS).^10^ Similarly, a meta-analysis showed intraoperative use of near-infrared fluorescent cholangiography with indocyanine green reduced the Bile Duct Injury (BDI) sizably.^15^ In case of a biliary injury, a study suggested that the repairs should be done either within 72 hours or delayed (6 weeks) after LC.^16^ And, repairs done in the intermediate period led to biliary stricture. Our previous study and multiple studies have shown that a single setting Endoscopic Retrograde Cholangiopancreatography (ERCP) with LC or early LC is feasible for symptomatic GSD with concomitant CBD stones.^2,17^ And, early LC following ERCP was associated with shorter operation time, fewer fibrotic changes in the gallbladder, and lower risk for the development of complications.

Our study had its limitations. It was a single-centre study without long-term follow-up. And it is difficult to generalize the result nationally. Also, an observational study without a control group has its own innate limitations such as causality and association could not be established.

## CONCLUSIONS

This study showed that the prevalence of open cholecystectomy among patients undergoing laparoscopic cholecystectomy was lower when compared to similar studies conducted in similar settings. With recent advances in the laparoscopic technique, conversion rate and complications like damage to major structures could be brought down to a minimum.
